# Shortening and Improving the Embryonic Stem Cell Test through the Use of Gene Biomarkers of Differentiation

**DOI:** 10.1155/2011/286034

**Published:** 2011-08-24

**Authors:** Andrea C. Romero, Eugenio Vilanova, Miguel A. Sogorb

**Affiliations:** Unidad de Toxicología y Seguridad Química, Instituto de Bioingeniería, Universidad Miguel Hernández de Elche, Avenida de la Universidad s/n, 03202 Elche, Spain

## Abstract

The embryonic Stem cell Test (EST) is a validated assay for testing embryotoxicity *in vitro*. The total duration of this protocol is 10 days, and its main end-point is based on histological determinations. It is suggested that improvements on EST must be focused toward molecular end-points and, if possible, to reduce the total assay duration. Five days of exposure of D3 cells in monolayers under spontaneous differentiation to 50 ng/mL of the strong embryotoxic 5-fluorouracil or to 75 **μ**g/mL of the weak embryotoxic 5,5-diphenylhydeantoin caused between 20 and 74% of reductions in the expression of the following genes: *Pnpla6*, *Afp*, *Hdac7*, *Vegfa*, and *Nes*. The exposure to 1 mg/mL of nonembryotoxic saccharin only caused statistically significant reductions in the expression of *Nes*. These exposures reduced cell viability of D3 cells by 15, 28, and 34%. We applied these records to the mathematical discriminating function of the EST method to find that this approach is able to correctly predict the embryotoxicity of all three above-mentioned chemicals. Therefore, this work proposes the possibility of improve EST by reducing its total duration and by introducing gene expression as biomarker of differentiation, which might be very interesting for *in vitro* risk assessment embryotoxicity.

## 1. Introduction

Toxicity to reproduction has to be mandatorily assessed in developed countries for the registration and authorization of all chemicals with medium and high production volumes. Toxicity to reproduction includes adverse effects of chemicals on fertility, embryotoxicity, teratogenicity, and development. OECD has its own *in vivo* guidelines for testing either teratogenicity (OECD 414) or toxicity to reproduction (testing simultaneously all the above-stated adverse effects) by performing two generation toxicity assays (OECD 416) [1]. It seems remarkable that there are no *in vivo* guidelines for testing embryotoxicity, which has to be assayed in OECD guideline 416 [2]. Assays according to OECD-416 guideline are time consuming and expensive as they involve lots of animals. It is estimated that one assay of this kind to test a single chemical requires 3200 animals [3] with an estimated cost of more than €300000 [4]. Thus, it is obvious that a fast, safe, and reliable *in vitro* alternative method for performing embryotoxicity risk assessment would be welcomed by industry. Furthermore, it would help to save lots of resources devoted to test other toxicity to reproduction effects accounting after embryonic development, which is especially relevant for the massive screenings performed during the early development stages of chemicals for lots of purposes (i.e., pharmaceuticals, cosmetics, biocides, food additives, etc.).

The only validated methods available for testing embryotoxicity *in vitro* are the embryonic stem cell test (EST) (see abbreviations), the mouse whole embryo culture, and the micromass method. Of these, EST is the only “pure” *in vitro* method because it totally suppresses the use of animals [2]. EST uses two mouse cell lines, D3 embryonic stem cells for testing embryotoxicity by monitoring general cytotoxicity and disturbances in their differentiation, and 3T3 fibroblasts embryonic cells for testing general cytotoxicity in a nondifferentiating system. EST uses three different end-points, IC_50_D3 and IC_50_3T3, which are defined as the concentration of the tested chemical that causes 50% reduction in the viability of D3 and 3T3 cells, respectively, and ID_50_, defined as the concentration of the tested chemical that causes inhibition of D3 cells differentiation by 50% [5]. These three end-points are further integrated into three linear discriminant functions which discriminate the embryotoxic potential of chemicals into three categories: non, weak, and strong embryotoxicity [6].

EST is relatively lengthy because it requires the exposure of cells to tested chemicals for 10 days. Its technical complexity is also relatively high because it includes cell cultures in monolayers to obtain IC_50_D3 and IC_50_3T3 through cytotoxicity assays, as well as the culture of “hanging drop” embryoid bodies (EBs) for testing alterations in the differentiation of D3 cells. EST is also laborious because it entails two changes of culture medium (of three different cultures containing the two different cell lines), which are specifically prepared with all 6–8 different concentrations of the tested chemical. Besides, more than 300 EBs per single test have to be individually seeded and further optically analyzed. Additionally, a main weakness of EST is the need to monitor changes in the D3 EBs differentiation caused by exposure to the assessed chemical. Indeed, it is performed by an individual microscopic inspection of the 10-day differentiated EBs to determine if they are contractile, or not, without considering other relevant circumstances such as the total beating area.

Despite the above-stated drawbacks, EST has overcome a blind interlaboratory validation study sponsored by the European Centre for Validation of Alternative Methods (ECVAM) that considers EST ready for the *in vitro* screening embryotoxicity of chemicals [7]. However, ECVAM also considers that EST is still not ready for regulatory purposes and that its performance has to be improved by adopting several approaches. In this way, some of ECVAM's suggestions to be implemented in the EST protocol [8] are to introduce molecular end-points (for a better quantification of D3 cells differentiation) and to introduce end-points for monitoring the differentiation to the three main embryonic lineages (and not only mesoderm-derived cardiomyocytes).

In this work, we followed ECVAM's suggestions by using the expression of several genes deriving from mesoderm, endoderm, and ectoderm as biomarkers of differentiation. We used these biomarkers to estimate ID_50_ based on molecular approaches. We found that the linear discriminating functions originating from the EST protocol were able to correctly predict the embryotoxicity of the strong embryotoxic chemical 5-fluorouracil (5-FU), the weak embryotoxic chemical 5,5-diphenylhydantoin (DPH), and the nonembryotoxic chemical saccharin. We also proposed other improvements to the EST protocol by employing monolayer cultures instead of hanging drops EBs and by cutting the exposure time from 10 days to 5 days.

## 2. Materials and Methods

### 2.1. Cell Cultures

D3 cells were grown on monolayers in undifferentiated state on 75-mm plates in Dulbecco's modified eagle medium (DMEM), medium supplemented with 15% heat-inactivated fetal bovine serum, 1% nonessential amino acids, 50 units of penicillin/mL, 100 *μ*g streptomycin/mL, 0.1 mM *β*-mercaptoethanol, and 1000 units of leukemia inhibition factor (LIF)/mL. Undifferentiated cells were incubated at 37°C in an atmosphere with 1.5% CO_2_ and 95% humidity. For culturing D3 cells under spontaneous differentiation, LIF was removed from the medium culture, and the CO_2_ concentration was increased to 5%.

3T3 cells were grown on monolayers on 75 mm plates in DMEM medium supplemented with 10% calf serum, 50 units of penicillin/mL, and 100 *μ*g streptomycin/mL. Cells were incubated at 37°C in an atmosphere with 5% CO_2_ and 95% humidity.

### 2.2. Cellular Exposures to Chemicals

Fresh 5-FU, DPH, or saccharin were added to the medium cultures at the appropriate concentrations just before starting exposures. D3 cells under spontaneous differentiation were cultured for 5 days on monolayers in P100 Petri dishes (for gene expression monitoring) or in 96-well plates (for cytotoxicity assays) as stated above. 3T3 cells were cultured in 96-well plates as described above and were also exposed to 5-FU, DPH, or saccharin for 5 days. In all cases, the culture medium was changed on day 3 of exposure.

### 2.3. Cytotoxicity Assays

The cytotoxicity caused by exposure to 5-FU, DPH, and saccharin was assayed with the 3-(4,5-dimethylthiazol-2-yl)-2,5-diphenyltetrazoliumbromide (MTT) assay, which is the cytotoxicity assay used in the EST protocol [6]. This test is based on the ability of mitochondrial dehydrogenases to convert the yellow substrate MTT into a dark blue formazan product. 

D3 and 3T3 cells were seeded at 20000 cells/well and incubated as described above in the presence of different 5-FU, DPH, or saccharin concentrations. On day 5 of exposure, chemicals were removed, and cells were incubated with 1 mg MTT/mL for 3 hours. At the end of this period, MTT was removed, and cells were washed with phosphate buffer saline. Finally, 100 *μ*L of dimethyl sulfoxide/well were added to lysate cells, and the generated formazan was monitored recording absorbance at 570 nm. It was assumed that control cultures (not exposed to 5-FU, DPH, or saccharin) presented maximum viability, and the results were expressed as a percentage as regards these controls. Each condition was assayed in 12 independent wells.

### 2.4. Quantification of the Gene Expression

The expression of the genes proposed as biomarkers of differentiation was recorded by quantitative RT-PCR with three independent plates per assayed condition.

Cell cultures were trypsinized after exposure to 5-FU, DPH, or saccharin, and total RNA was extracted with Tripure according to Chomczynski and Sacchi [9]. RNA was quantified and purity determined according to the 260/280 nm optical density ratio. One *μ*g of RNA was reverse transcribed using Expand Reverse Transcriptase and oligo-dT primers (Roche) according to the manufacturer's recommendations.

All the genes (except *Pnpla6*) were assayed using the Light Cycler Fast Start DNA Master PLUS SYBR Green I kit (Roche). The PCR program consisted in an initial step at 95°C for 10 minutes followed by 40 cycles of 10 seconds denaturing at 95°C, 7 seconds at the respective annealing temperature, and 12 seconds at 72°C, plus a final melting curve step.  Table 1 indicates the primer sequences and the annealing temperatures employed for each gene. The *Pnpla6 *expression was monitored in the samples analyzed with the specific Taqman kit supplied by Applied Biosystems.

Quantification was performed by considering 2^(−ΔΔCt)^ calculations [10] with the Step-One software, v2.0.1 (Applied Biosystem). **β*-Actin* was used as an invariant internal control for each sample.

### 2.5. Results Validation

The performance of the proposed end-points was validated by applying the same linear discriminant functions and by following the classification criteria used in the EST protocol. These functions and criteria are summarized in Table 2.

### 2.6. Statistical Significance

Differences between the gene expression of the control and chemical-exposed cultures were statistically analyzed with Student's *t*-tests performed with Graph-Pad Instat (v 3.06). The level of significance (*P*) is indicated in each case.

## 3. Results

### 3.1. Cytotoxicity of 5-FU, DPH, and Saccharin

**Figure 1:**
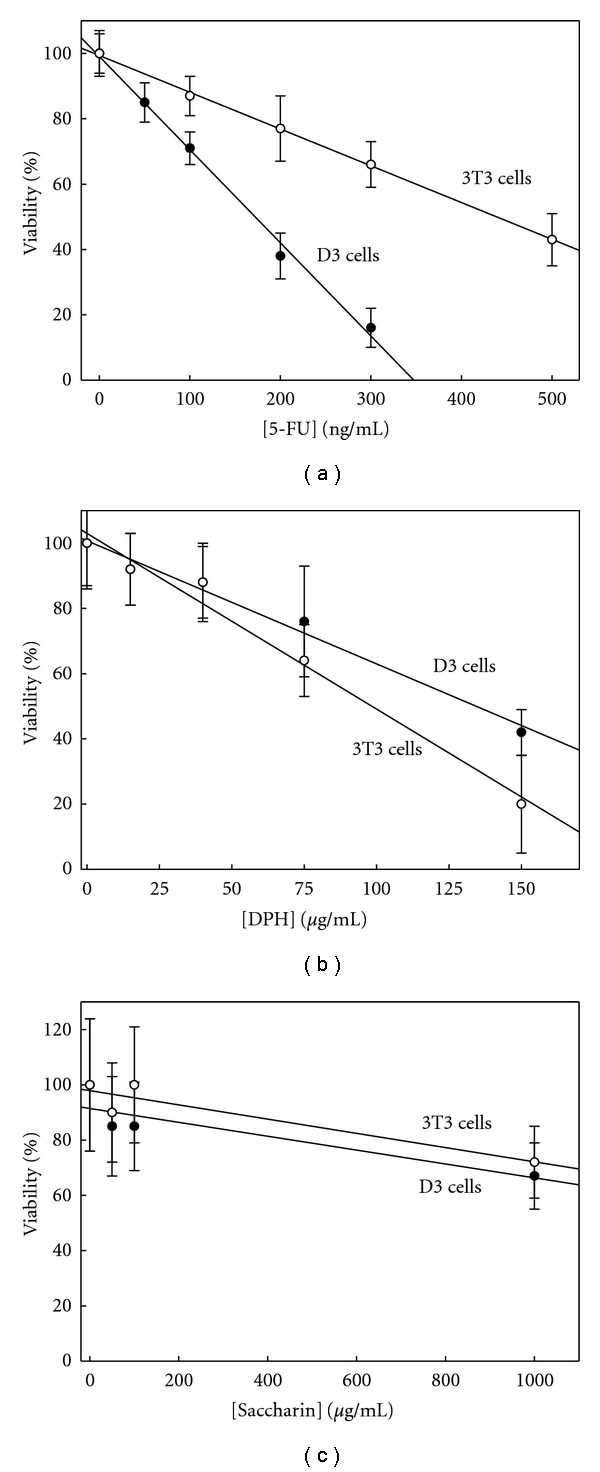
Effect of 5-FU, DPH, and saccharin on cell viability. D3 and 3T3 cells were cultured on monolayers exposed to 5-FU (a), DPH (b), or saccharin (c) for 5 days. Then the MTT test was performed as described in Section 2. D3 cells were cultured under spontaneous differentiation (in the absence of LIF). The results are expressed as a percentage of absorbance at 570 nm with regards to the control cultures, which were not exposed to 5-FU, DPH, or saccharin. Each condition was assayed with twelve independent wells.

The cytotoxicity caused by 5-FU, DPH, and saccharin after 5 days of exposure to D3 and 3T3 cells was assayed with the MTT test since these end-points are required to assay embryotoxicity according to EST. 5-FU was more toxic for D3 cells than for 3T3 cells (Figure 1(a)). IC_50_D3 was 0.17 *μ*g/mL, while IC_50_ for 3T3 cells was recorded to be 2.6 times higher (Table 3). We chose the 50 ng/mL concentration to test the effect of 5-FU on several biomarkers of differentiation which, according to Figure 1(a), would cause a reduction of the viability for D3 and 3T3 cells by 15% and 6%, respectively. 

On the opposite to 5-FU, the cytotoxicity of DPH was higher (1.4 times) for 3T3 than for D3 cells (Figure 1(b), Table 3). We studied gene expression on D3 cells exposed for 5 days to 75 *μ*g DPH/mL, which according to Figure 1(b) would cause loss of viability of 28%. The estimated reduction of 3T3 cell viability caused by this exposure was 38%. 

The IC_50_ for D3 and 3T3 cells of saccharin were both higher than 1000 *μ*g/mL (Figure 1(c), Table 3). This concentration (1000 *μ*g/mL) was used for testing negative controls in the standard EST tests and was selected for testing its effects on gene expression of D3 cells under spontaneous differentiation.

### 3.2. Expression of Gene Markers of Differentiation on D3 Cells Exposed to 5-FU, DPH, and Saccharin

**Figure 2:**
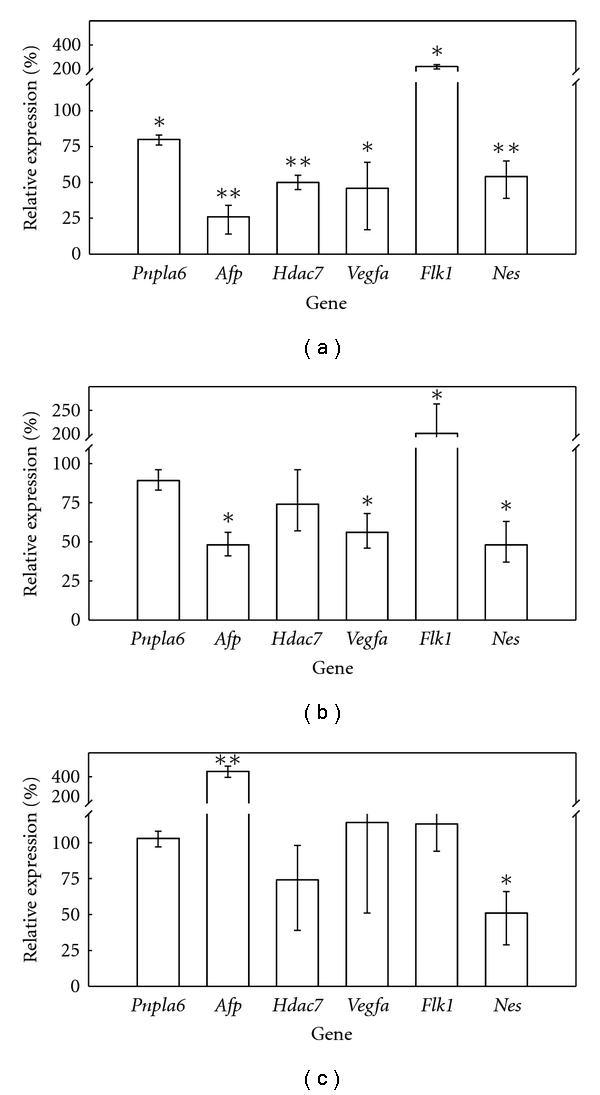
Effect of 5-FU, DPH, and saccharin on D3 differentiation. Monolayer cultures of D3 cells under spontaneous differentiation (in the absence of LIF) were exposed to either 50 ng 5-FU/mL (a), or 75 *μ*g DPH/mL (b), or 1000 *μ*g saccharin/mL (c) over 5 days. When exposure ended, RNA was extracted, and the gene expression of the biomarkers of differentiation was assayed by quantitative RT-PCR according to the procedure described in Section 2. The gene expression was expressed as a percentage as regards the control cultures (not exposed to chemicals). Each condition (control and exposed cultures) was assayed in three independent plates. (∗: statistically different from controls for *P *< 0.05; ∗∗: statistically different from controls for *P* < 0.01).

We assayed the expression of several genes considered useful as biomarkers of differentiation to propose molecular end-points for monitoring the changes taking place in the process as a result of exposure to embryotoxic chemicals, which would potentially improve the microscopic observation of beating EBs. We found (Figure 2(a)) that the exposure of D3 cells under spontaneous differentiation to 50 ng 5-FU/mL for 5 days inhibited the expression of patatin-like phospholipase domain containing 6 (*Pnpla6*), *α*-fetoprotein (*Afp*), histone deacetylase 7 (*Hdac7*), vascular endothelial growth factor A (*Vegfa*), and nestin (*Nes*) by 20, 74, 50, 54, and 46%, respectively. It is also remarkable to note that the expression of fetal liver kinase 1 (*Flk1*) was also altered but, in this case, we detected an increase in the expression of 2.2 times (Figure 2(a)) instead of inhibition. In all cases, the changes in the expression were statistically significant (Figure 2(a)). Thus, we can estimate that the 5-FU concentrations causing a 50% reduction in the expression of the assayed biomarkers (which could be considered the equivalent to the ID_50_ end-point in EST) would be around 50 ng/mL for *Hdac7*, *Vegfa*, and *Nes*, higher (but in the same order of magnitude) than 50 ng/mL for *Pnpla6* and lower than 50 ng/mL for *Afp* (Figure 2, Table 3). 

The exposure (5 days to 75 *μ*g/mL) to DPH of D3 differentiating cells reduced the expression of *Pnpla6*, *Afp*, *Hdac 7*, *Vegfa*, and *Nes* to 89, 48, 74, 56, and 48%, respectively, of nonexposed differentiating cells (Figure 2(b)). DPH also increased expression of *Flk 1* in a similar extension (2.1 times) as 5-FU did (Figure 2(b)). These changes in the gene expression were all statistically significant except for *Pnpla6* and *Hdac7*. These records allow estimating that concentrations of DPH causing reduction of 50% in the expression of *Afp*, *Nes*, and *Vegfa* would be around 75 *μ*g/mL, and slightly higher for *Pnpla6* and *Hdac7*.

The exposure of D3 cells to 1000 *μ*g saccharin/mL during 5 days causes no significant alterations in the expression of *Pnpla6*, *Vegfa*, and *Flk1*, reductions in the expression of *Hdac7* (26%) and *Nes* (49%), and a strong increase (4.4 times) in the expression of *Afp* (Figure 2(c)).

### 3.3. Tested Biomarkers Ability to Assign Embryotoxic Potential

We validated the expression of our biomarker genes as regards their ability to detect and classify embryotoxic chemicals using the same linear discriminant functions employed in EST with ID_50_ as the 5-FU, DPH, and saccharin concentrations which inhibit the expression of the gene by 50% (Table 3). We found that *Pnpla6*, *Afp*, *Vegfa*, *Hdac7*, and *Nes* were able to assign the label of strong embryotoxicant to 5-FU and weak embryotoxicant to DPH, in accordance with its *in vivo* toxicity, and also as was previously reported with standard EST [5]. *Pnpla6*,* Flk1*,* Vegfa*, *Hdac7*, and *Nes* also properly labeled saccharin as nonembryotoxic chemical (Table 3).

## 4. Discussion

We tested the expression of the biomarker genes of early differentiation as possible end-points to improve EST performance. At the same time, we proposed a reduction in the total test duration and a simplification of the process since we used monolayer cultures instead of “hanging drops” EBs. The implementation of changes to EST would refine its predictability and allow its future use for regulatory purposes, which would prove useful for the risk assessment of embryotoxicity, and subsequently for toxicity to reproduction.

### 4.1. Cytotoxicity

We found that 5-FU was 2.6 times more toxic to D3 cells than to 3T3 cells (Table 3), which was expected since 5-FU was classified as a strong embryotoxicant. These results are comparable to those found in the EST validation study sponsored by ECVAM, where 5-FU was found to be 3.3 times more toxic for D3 cells than for 3T3 cells [6]. This slight difference can be justified on the basis of differences in the exposure (10 days in the case of the EST validation study and 5 days in our work). This difference also justifies that our recorded IC_50_ was 1.8 and 1.5 times higher for D3 and 3T3, respectively, than the values recorded with the standard EST protocol [6]. The IC_50_ found in this study for 3T3 cells exposed to DPH was 1.7 times higher than the record obtained in the ECVAM's EST validation study [6], which is in concordance with differences found in the case of 5-FU. The cytotoxicity records found for D3 and 3T3 cells exposed to saccharin were also consistent with published results because we also found figures higher than 1000 *μ*g/mL [6].

### 4.2. Expression of Biomarkers of Differentiation

*Pnpla6* is the gene that codifies for a protein called neuropathy target esterase (NTE). NTE is a target of a neurodegenerative syndrome caused by certain organophosphorus pesticides. Additionally, homozygous NTE^−/−^ mice embryos were not viable due to failures in vasculogenesis [12], and although heterozygous NTE^+/−^ mice embryos were viable, the resulting animals displayed alterations in the nervous system [13]. We previously reported that *Pnpla6* is expressed constitutively in D3 cells although a peak in expression is reached in initial differentiation stages [14]. All these data indicate that *Pnpla6* may play critical roles in normal nervous system development (ectoderm-derived tissue) and blood vessels (endoderm-derived tissues), suggesting that this gene is a good candidate to be used as a biomarker of early differentiation. We found that 5-FU and DPH caused reductions (statistically significant in the case of 5-FU) in the expression of *Pnpla6* under our assay conditions (Figures 2(a) and 2(b)), indicating that *Pnpla6* might be used for detecting exposure to embryotoxic chemicals by monitoring changes in differentiation.

*Afp* is typically considered a biomarker of the visceral endoderm [15]. Exposure of D3 cells under differentiation to 50 ng 5-FU/mL and to 75 *μ*g DPH/mL inhibited the *Afp* expression by 74% (Figure 2(a)) and 52% (Figure 2(b)), respectively, which suggests that *Afp* is a very sensitive biomarker of exposure to embryotoxicants. These results are supported by other previous reports which found that the expression of this gene was inhibited in 20-day-old EBs exposed to 5-FU concentrations several orders of magnitude lower than IC_50_ [16]. In our case, we have demonstrated that *Afp's* sensitivity as a biomarker is maintained, even only after 5 days of differentiation.

*Vegfa* and *Hdac7* are considered endoderm biomarkers since the former is involved in the vasculogenesis of yolk sac and hematopoiesis [17], while the latter is implicated in vasculogenesis, endothelial cells migration, and vascular integrity maintenance [18]. The expression of both genes was inhibited by around 50% after 5 days of exposure to 5-FU or DPH (Figures 2(a) and 2(b)), suggesting that both genes can be used for our purposes. The expression of *Vegfa* has also been previously proposed as a molecular end-point for EST [19], although in our case, we cut the exposure time by half to demonstrate that the gene maintains its good performance as a biomarker of differentiation. To our knowledge, this is the first proposal of *Hdac7* as a biomarker of differentiation to be used in EST.

*Nes* codifies for neurofilament proteins and, therefore, plays a critical role in maintaining cellular integrity [20]. We found a statistically significant reduction in the expression of *Nes* as a result of cell exposure to 5-FU, DPH, and saccharin (Figure 2), which indicates that *Nes* might be a good biomarker of exposure to embryotoxicants, at least in D3 cells, as they displayed good compromise in differentiation toward the neuroectoderm when cultured in monolayers [21]. 

*Flk1* is an early mesodermal biomarker [22], which is also altered by 5-FU exposure (Figure 2). However, unlike other assayed biomarkers, 5-FU and DPH led to increases of expression instead of inhibitions (Figures 2(a) and 2(b)), which means that *Flk1* is unsuitable for use in accordance with the EST approach since its mathematical discriminant function was developed considering end-points which were underexpressed as regards the control and not overexpressed as in *Flk1* in our study. Nevertheless, changes in the expression of *Flk1* were statistically significant, and this information can be valuable as it complements the information obtained for other biomarkers.

### 4.3. Performance of Biomarkers for Discrimination of Embryotoxicant Potency

5-FU is a well-known *in vivo* embryotoxic agent that has been labeled by EST as a strong embryotoxicant (it is included as a positive control in the standard EST protocol). We applied the mathematical functions of EST to our results by considering ID_50_ as the value of the 5-FU concentration that inhibited the expression of each biomarker by 50% to determine if our proposed approach yields the same results as the standard protocol. We found that *Vegfa*, *Hdac7*, and *Nes* predicted that 5-FU was a strong embryotoxicant (function III > function I and function III > function II) (Table 3). We could not estimate ID_50_ for *Pnpla6* but, according to Figure 2(a), we can establish that it is higher than 50 ng/mL. The analysis done of the discriminant functions with the available data demonstrated that the conditions to be labeled as strong embryotoxic (function III > function I and function III > function II) were lost only for an ID_50_ higher than 1.5 *μ*g/mL. Figure 1(a) illustrates that this exposure would cause total loss of the viability of both the D3 and 3T3 cells, therefore suggesting that if ID_50_ for *Pnpla6* was determined, then 5-FU would also be labeled as a strong embryotoxicant. The ID_50_ for *Afp* could not be determined with the available data, but it is concluded that it must be lower than 50 ng/mL (Figure 2(a)). However, with the available information, the analysis of the discriminant functions once again demonstrated that if ID_50_ < 50 ng/mL, then necessarily III > II and III > I; therefore, 5-FU was correctly classified as a strong embryotoxicant when using *Afp* as a biomarker of differentiation.

DPH is a well-known *in vivo* embryotoxic agent that has been labeled by EST as a weak embryotoxicant. When we applied the above-explained approach, we found that in the case of *Nes*, *Vegfa*, and *Afp*, function II was always higher than functions I and II (Table 3), which led to necessarily classify DPH as weak embryotoxicant as EST does. The DPH concentrations with capability to inhibit 50% of the expression of *Pnpla6* and *Hdac7* could not be exactly estimated but according to Figure 2(b) should be in the same order of magnitude as 75 *μ*g/mL, which according to IC_50_ records yields the functions I, II, and III as was stated in Table 3. In this situation and taking into consideration that ID_50_ should necessarily be positive, II always will be higher than III, and II will be higher than I (the needed condition to label DPH as weak embryotoxicant) for ID_50_ < 143 *μ*g/mL, which is compatible with data displayed in Figure 2(b) where we demonstrated that 75 *μ*g DPH/mL was able to inhibit 11 and 26% of *Pnpla6* and *Hdac7* expression, respectively.

The expression of *Nes* was inhibited by 50% by the exposure to 1000 *μ*g saccharin/mL (Figure 2(c)). Considering the records for IC_50_, we found that saccharin must be classified as nonembryotoxicant, since I > II and I > III (Table 3). We also found that ID_50_ for *Pnpla6*, *Hdac7*, *Vegfa*, *Flk1*, and *Nes* was always higher than 1000 *μ*g saccharin/mL, which becomes EST discriminant functions in equations showed in Table 3. Considering that ID_50_ must be necessarily positive, it supposes that I will be always higher than III and higher than II for ID_50_ higher than 420 *μ*g/mL, which is consistent to results shown in Figure 2(c) and led to necessarily classify saccharin as nonembryotoxicant, as was established by the EST method.

## 5. Conclusions

We propose implementing the expression of 6 different biomarkers of differentiation to EST end-points, which would improve the performance of this method for risk assessment (specifically in the step of hazard identification). These biomarkers have successfully predicted the embryotoxicity of 5-FU, DPH, and saccharin, but an assessment with another embryotoxicant chemicals is pending. In addition, we introduced three other improvements: reducing the total test time by half, employing monolayer cultures instead of more technically complex techniques to manage EBs, and the possibility to assess several end-points (the expression of several genes) for the same exposed cells.

## Figures and Tables

**Table 1:** Primer sequences and annealing temperatures used in the quantitative RT-PCR experiments.

Gene	5′–3′ oligo	3′–5′ oligo	T (°C)
*Actin*	CCCTAGGCACCAGGGTGTGA	TCCCAGTTGGTAACAATGCCA	62
*Afp*	GCTGCAAAGCTGACAACAAG	GGTTGTTGCCTGGAGGTTTC	63
*Vegfa *	CGTTCACTGTGAGCCTTGTTCAG	GCCTTGCAACGCGAGTCTGT	60
*Hdac7*	CCATGTTTCTGCCAAATGTTTTGG	GCCGTGAGGTCATGTCCACC	63
*Flk1*	CAGCCAGACAGACAGTGGGATGGTC	CCGAGGCCACAGACTCCCTGCTT	61
*Nes*	GCTTTCCTGACCCCAAGCTG	GGCAAGGGGGAAGAGAAGGA	61

**Table 2:** The linear discriminant functions and classification criteria considered in EST for assessing the embryotoxicity potential of chemicals. Data taken from [11]. IC_50_D3 was defined as the concentration that reduces the viability of D3 cells by 50% after 10 days of exposure. IC_50_3T3 was defined as the concentration that reduces the viability of 3T3 cells by 50% after 10 days of exposure. ID_50_ was defined as the concentration that inhibits the spontaneous differentiation of D3 embryoid bodies to contractile cardiomyocytes by 50%. IC_50_D3, IC_50_3T3, and ID_50_ must necessarily be expressed in *μ*g/mL.

I = 5.916 log(IC_50_3T3) + 3.500 log(IC_50_D3) − 5.307[(IC_50_3T3 − ID_50_)/IC_50_3T3] − 15.27		
II = 3.651 log(IC_50_3T3) + 2.394 log(IC_50_D3) − 2.033[(IC_50_3T3 − ID_50_)/IC_50 _3T3] − 6.85		
III = −0.125 log(IC_50_3T3) − 1.917 log(IC_50_D3) + 1.500[(IC_50_3T3 − ID_50_)/IC_50_3T3] − 2.67		

Strong embryotoxic if	Weak embryotoxic if	Non embryotoxic if
III > I and III > II	II > I and II > III	I > II and I > III

**Table 3:** Biomarkers validation. The capability of the tested biomarkers to assign an embryotoxic potential to chemicals was validated using the linear discriminate functions and the EST protocol criteria according to what Table 2 displays. IC_50_D3 and ID_50_3T3 records were obtained from Figure 1, while ID_50_ was obtained from Figure 2.

		IC_50_D3	ID_50_3T3	ID_50_	Linear discriminant functions			
Substance	Gene	(*μ*g/mL)	(*μ*g/mL)	(*μ*g/mL)	I	II	III	CLA
5-FU								

	*Pnpla6*	0.17	0.44	>0.05	−25 + 12ID_50_	−12 + 4.6ID_50_	0.37 − 3.4ID_50_	S
	*Afp*	0.17	0.44	<0.05	−25 + 12ID_50_	−12 + 4.6ID_50_	0.37 − 3.4ID_50_	S
	*Hdac7, Vegfa, Nes*	0.17	0.44	0.050	−25	−12	0.18	S
	*Flk1*	0.17	0.44	NA	—	—	—	NA

DPH								

	*Nes, Vegfa, Afp*	130	97	75	2.6	5.0	−6.6	W
	*Pnpla6, Hdac7*	130	97	>75	−1.4 + 0.05ID_50_	3.5 + 0.02ID_50_	−5.5 − 0.02ID_50_	W
	*Flk1*	130	97	NA	—	—	—	NA

Saccharin								

	*Nes*	1850	1640	1000	13	12	−8	NON
	*Pnpla6, Hdac7, Vegfa, Flk1, Nes*	1850	1640	>1000	10 + 0.003ID_50_	11 + 0.001ID_50_	−7.8 − 0.0008ID_50_	NON
	*Afp*	1850	1640	NA	—	—	—	NA

CLA: classification of embryotoxicity; S: strong embryotoxicant; W: weak embryotoxicant; NON: nonembryotoxicant; NA: not applicable.

## Abbreviations

5-FU: 5-fluorouracil
*Afp*: *α*-fetoprotein
DMEM: Dulbecco's modified eagle medium
DPH: 5,5-diphenylhydantoin
EBs: Embryoid bodies
ECVAM: European Centre for Validation of Alternative Methods
EST: Embryonic stem cell test
*Flk1*: Fetal liver kinase 1
*Hdac7*: Histone deacetylase 7
LIF: Leukemia inhibition factor
MTT: 3-(4,5-dimethylthiazol-2-yl)-2,5-diphenyltetrazolium bromide
*Nes*: Nestin
NTE: Neuropathy target esterase
*Pnpla6*: Patatin-like phospholipase domain containing 6
*Vegfa*: Vascular endothelial growth factor A.
